# FocAn: automated 3D analysis of DNA repair foci in image stacks acquired by confocal fluorescence microscopy

**DOI:** 10.1186/s12859-020-3370-8

**Published:** 2020-01-28

**Authors:** Simon Memmel, Dmitri Sisario, Heiko Zimmermann, Markus Sauer, Vladimir L. Sukhorukov, Cholpon S. Djuzenova, Michael Flentje

**Affiliations:** 1 Department of Radiation Oncology, University Hospital Würzburg, Josef-Schneider-Strasse 11, 97080 Würzburg, Germany; 2 Lehrstuhl für Biotechnologie und Biophysik, Biozentrum, Universität Würzburg, 97074 Würzburg, Germany; 3 Fraunhofer Institute for Biomedical Engineering (IBMT), Joseph-von-Fraunhofer-Weg 1, 66280 Sulzbach, Germany; 4 Molekulare und Zellulare Biotechnologie/Nanotechnologie, Universität des Saarlandes, Campus Saarbrücken, 66123 Saarbrücken, Germany; 5 Marine Sciences, Universidad Catolica del Norte, Casa Central, Angamos 0610, Antafogasta/Coquimbo, Chile

**Keywords:** DNA double-strand breaks, ImageJ plugin, γH2AX-foci, Automated analysis, Ionizing radiation, Open-source tool, Radiation biology

## Abstract

**Background:**

Phosphorylated histone H2AX, also known as γH2AX, forms μm-sized nuclear foci at the sites of DNA double-strand breaks (DSBs) induced by ionizing radiation and other agents. Due to their specificity and sensitivity, γH2AX immunoassays have become the gold standard for studying DSB induction and repair. One of these assays relies on the immunofluorescent staining of γH2AX followed by microscopic imaging and foci counting. During the last years, semi- and fully automated image analysis, capable of fast detection and quantification of γH2AX foci in large datasets of fluorescence images, are gradually replacing the traditional method of manual foci counting. A major drawback of the non-commercial software for foci counting (available so far) is that they are restricted to 2D-image data. In practice, these algorithms are useful for counting the foci located close to the midsection plane of the nucleus, while the out-of-plane foci are neglected.

**Results:**

To overcome the limitations of 2D foci counting, we present a freely available ImageJ-based plugin (FocAn) for automated 3D analysis of γH2AX foci in z-image stacks acquired by confocal fluorescence microscopy. The image-stack processing algorithm implemented in FocAn is capable of automatic 3D recognition of individual cell nuclei and γH2AX foci, as well as evaluation of the total foci number per cell nucleus. The FocAn algorithm consists of two parts: nucleus identification and foci detection, each employing specific sequences of auto local thresholding in combination with watershed segmentation techniques. We validated the FocAn algorithm using fluorescence-labeled γH2AX in two glioblastoma cell lines, irradiated with 2 Gy and given up to 24 h post-irradiation for repair. We found that the data obtained with FocAn agreed well with those obtained with an already available software (FoCo) and manual counting. Moreover, FocAn was capable of identifying overlapping foci in 3D space, which ensured accurate foci counting even at high DSB density of up to ~ 200 DSB/nucleus.

**Conclusions:**

FocAn is freely available an open-source 3D foci analyzer. The user-friendly algorithm FocAn requires little supervision and can automatically count the amount of DNA-DSBs, i.e. fluorescence-labeled γH2AX foci, in 3D image stacks acquired by laser-scanning microscopes without additional nuclei staining.

## Background

DNA double-strand breaks (DSBs) are biologically the most significant lesions produced by ionizing radiation (IR) and other exogenous cytotoxic agents. DSBs are the major threats to the genomic integrity of cells [1, 2] and if insufficiently repaired or misrepaired, DSBs may lead to chromosome breaks, deletions and translocations [3]. The physiological target of IR is not DNA itself but rather DNA in the context of chromatin, i.e. within a complex and highly regulated protein-DNA structure [4, 5]*.* It is well known that histone H2AX becomes phosphorylated at Serine139 to γH2AX immediately after irradiation, and involves a large chromatin region of up to ~ 2 Mbp, thus forming distinct μm-sized foci at the sites of DSBs [6]. γH2AX foci indicate sites of DSBs [7]. Therefore, the DNA DSBs can be visualized and quantified by fluorescence microscopy using antibodies recognizing γH2AX. H2AX phosphorylation recruits various DNA-damage repair (DDR) proteins to the DSB sites, which can also form foci that usually colocalize with γH2AX [8, 9].

Automated computer-based systems, which are able to evaluate large batches of image data uniformly, are gradually replacing the labor-intensive and bias−/error-prone method of manual foci counting [10]. Commercial software packages for the analysis of γH2AX are available either in combination with hardware, such as fully automatic microscope systems [11], stand-alone applications or macros [12–19]. Various signal thresholding and morphological algorithms applied to fluorescence images enable the accurate detection of nuclei and foci. In particular, image segmentation by watershed transformation algorithms allows to separate partially overlapping nuclei and foci [20]. However, most of the available automated foci counters were developed for 2D epi-fluorescence microscopy with poor axial (z) resolution. The counting is therefore performed in the midsection of the nucleus thereby neglecting the foci lying above and below the imaging plane.

In contrast, confocal microscopes are capable of imaging cell nuclei in 3D, typically with an axial resolution of about 400–800 nm and a lateral resolution of about 200 nm [21, 22]. Considering that the typical diameters of γH2AX foci are ~ 0.5–1 μm (i.e. above the resolution limit) [23], confocal microscopy is well suited for 3D analysis and quantification of DNA DSBs. Recently, the 3D image reconstruction approach based on the commercial Imaris Image Analysis software has been successfully applied to analyze DNA DSBs in various human cell types [24]. Currently, there are only a few free open-source software packages available, e.g. FociPicker, FindFoci and CellProfiler, which are capable of processing 3D image stacks for DSB foci counting [19, 25, 26].

Here we introduce a new algorithm for foci analysis (FocAn) capable of automatic 3D recognition of the total number of γH2AX foci per cell nucleus. FocAn is an easy-to-use and user-friendly software based on the open-source platform ImageJ. Another advantage of FocAn is its ability to recognize cell nuclei without additional nucleus staining (e.g. DAPI or Hoechst 33342 dyes), which is necessary for most established approaches [11–16]. This is achieved by using specific sequences of auto local thresholding in combination with watershed segmentation techniques.

### Experimental and computational methods

#### Cell culture

DK-MG and SNB-19 cell lines were obtained from DSMZ (Braunschweig, Germany) and routinely cultured in Dulbecco’s modified Eagle’s medium (DMEM, Sigma, Deisenhofen, Germany) complete growth medium (CGM) supplemented with 10% FCS under standard growth conditions (5% CO_2_, 37 °C). For experiments, cells were cultured on glass slides up to subconfluency.

#### X-ray irradiation

In preliminary experiments we irradiated cells with different single doses (1, 2, 3, 4 Gy) and found that above 2 Gy the number of γH2AX foci per nucleus was too high to discriminate single foci. Besides this, a typical treatment scheme for glioblastoma is 1.8 to 2.0 Gy single daily fraction to a whole therapy dose of 60 Gy. For that reason, in the present study we irradiated glioblastoma cells with 2 Gy, which is a therapeutically relevant dose of ionizing radiation. Irradiation was performed at room temperature using a 6 MV Siemens linear accelerator (Siemens, Concord, CA) at a dose rate of 2 Gy/min. After irradiation, cells were kept in CGM for the indicated time until fixation with paraformaldehyde.

#### Antibodies

The primary antibody used for labelling was a mouse monoclonal anti-phospho-histone H2A.X (Ser139) (γH2AX, Merck, Darmstadt, Germany). The secondary antibody was a Alexa647 conjugated F (ab)2 goat anti-mouse antibody (ThermoFischer Scientific, Schwerte, Germany). Both antibodies were diluted (1:400) in phosphate buffered saline (PBS) containing 5% bovine serum albumin (BSA).

#### Fluorescence staining of γH2AX and image acquisition

Cells were cultured on glass slides to subconfluency, and fixated as described previously [27]. Fixed cells were permeabilized with 0.5% Triton X-100 solution in PBS and then incubated with γH2AX antibodies for 2 h at room temperature. After washing 3 times with PBS containing 0.01% TWEEN20, the cells were incubated with secondary Alexa647 conjugated antibodies for 2 h at room temperature and washed 3 times with PBS containing 0.01% TWEEN20. Confocal image stacks were acquired with a Zeiss LSM 700 microscope using a voxel size of 0.1 × 0.1 × 0.25 μm.

#### Local thresholding in ImageJ

As already mentioned, FocAn is capable of identifying cell nuclei without using DNA binding fluorophores. Instead, nuclei recognition relies on the fluorescence of immunostained γH2AX as well as on the dim background fluorescence of the nuclei. To this end, the FocAn algorithm uses the auto local threshold ImageJ plugin available in the ImageJ depository (https://imagej.net/Auto_Local_Threshold). Prior to processing the images are reduced to 8-bit in order to increase the processing speed. Our plugin then binarizes 8-bit images using various local thresholding methods, which transforms each pixel according to the image characteristics within a domain of radius *r* (in pixel units) around the pixel. FocAn consecutively applies three thresholding methods (Eqs. 1–3):

1 $$ p=\left(\ p> mean-c\ \right)\kern1em ?\kern1em Object: Background, $$

2 $$ p=\left(\ p>\left(\frac{\left(\ \mathit{\max}+\mathit{\min}\ \right)}{}2\ \right)-c\ \right)? Object: Background, $$

3 $$ p=\left(\ p> median-c\ \right)\kern0.75em ? Object: Background, $$

where *p* stands for an analyzed pixel with an 8-bit gray value. The local domain operators *mean, median* and (*max + min*)/2 select the threshold, respectively, as the mean, median and mid-gray of the local grayscale distribution within the local domain radius around the analyzed pixel. The parameter *c* (default *c* = 0) can be used to manually adjust the applied threshold. If the *p*-value is larger than the local domain operator of the area surrounding the pixel (i.e. above the threshold), the pixel value will be set to zero (black). Otherwise the pixel value is set to 255 (white). As a result, binary (i.e. black and white) images are generated. The parameter “mean nucleus diameter”, prompted in the main window of the graphical user interface (GUI, Additional file 1: Figure S1A), is used to calculate the local domain radius. However, in cell types with low non-specific γH2AX staining in the nucleus, such as quiescent peripheral blood lymphocytes [28, 29], DNA staining fluorophores would have to be used to ensure nuclei recognition.

### Implementation

**Fig. 1:**
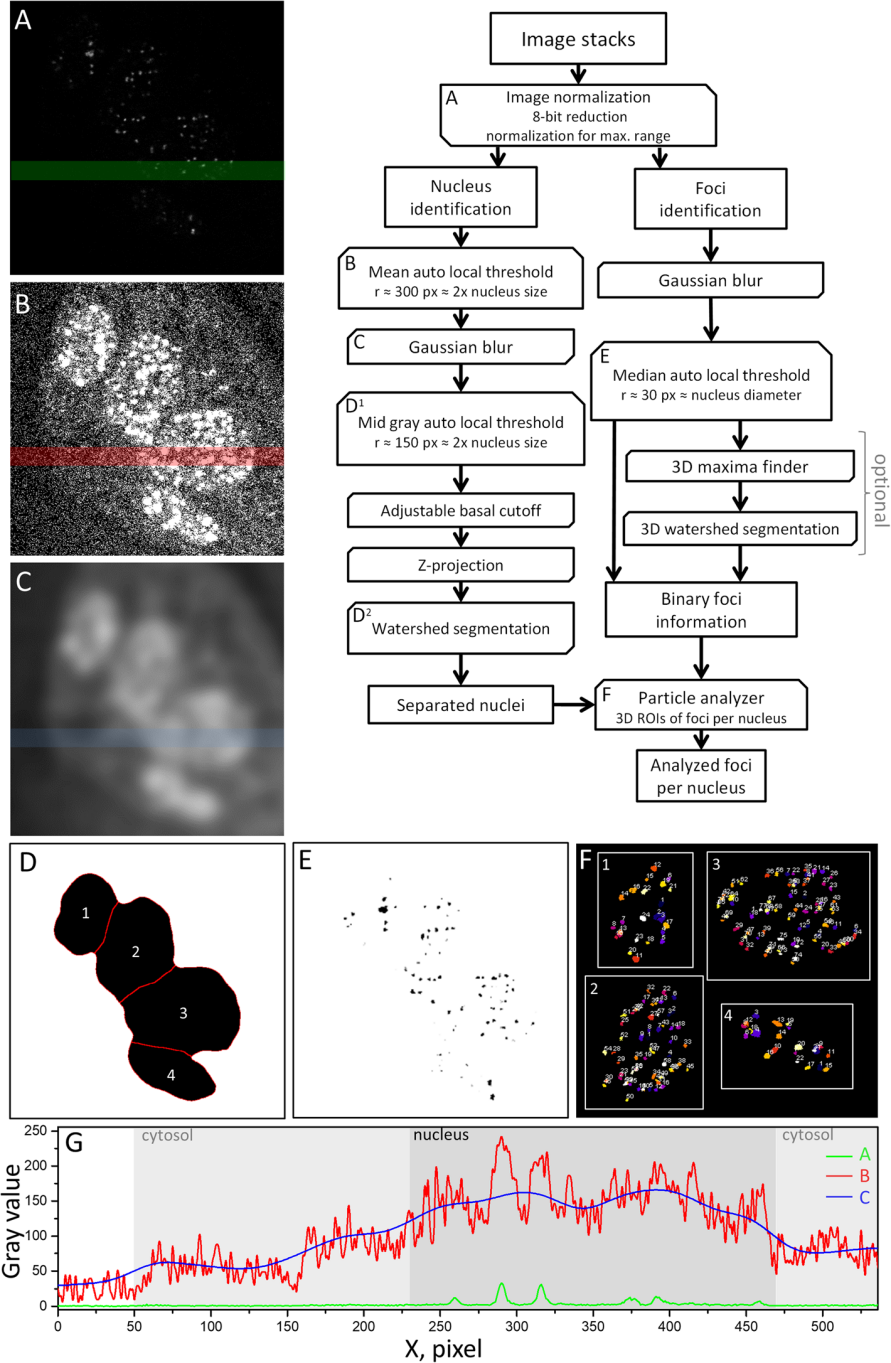
Flow chart demonstrating the main steps of the FocAn algorithm consisting of two independent components for nuclei (**b-d**) and foci (**e-f**) identification. In the first step (A), the raw image is normalized. Nucleus identification is then performed using a mean auto local threshold (ALT, B) followed by Gaussian blurring (**c**). Together, these steps result in gradual signal separation of nuclear and cytosolic areas (**c**), which is also illustrated in (**g**). The green, red and blue lines in (**g**) represent the intensity profiles of the corresponding colors in (**a**), (**b**) and (**c**), respectively. After that, mid-gray ALT creates a binary image, shown in (**d**). This is followed by watershed transformation for separation of overlapping nuclei, encircled with red lines in (**d**). The foci identification process starts with Gaussian blurring of the normalized images followed by median ALT (**e**). A 3D watershed transformation can be performed optionally, before finally the foci numbers per nucleus are determined (**f**)

FocAn is written in Ij1 macro language and runs on the open-source software ImageJ (v1.51 or above) on Windows, Linux or macOS systems. The source code is available at https://sourceforge.net/projects/focan-3d/files/ and is schematically outlined in Fig. 1. The algorithm is designed to analyze multiple image stacks successively. The main steps of FocAn include recognition of nuclei (Figs. 1b-d) followed by recognition of γH2AX foci (Figs. 1e-f).

In the first step (Fig. 1a, “image normalization”), raw image stacks are converted to 8-bit and normalized with zero saturation for each image slice separately. Normalization is necessary to compensate for photobleaching-related signal losses between subsequent slices. The immunostained γH2AX foci are clearly seen in the normalized image while the unstained cell nuclei (i.e. nuclear area) are barely visible (Fig. 1a).

**Fig. 2:**
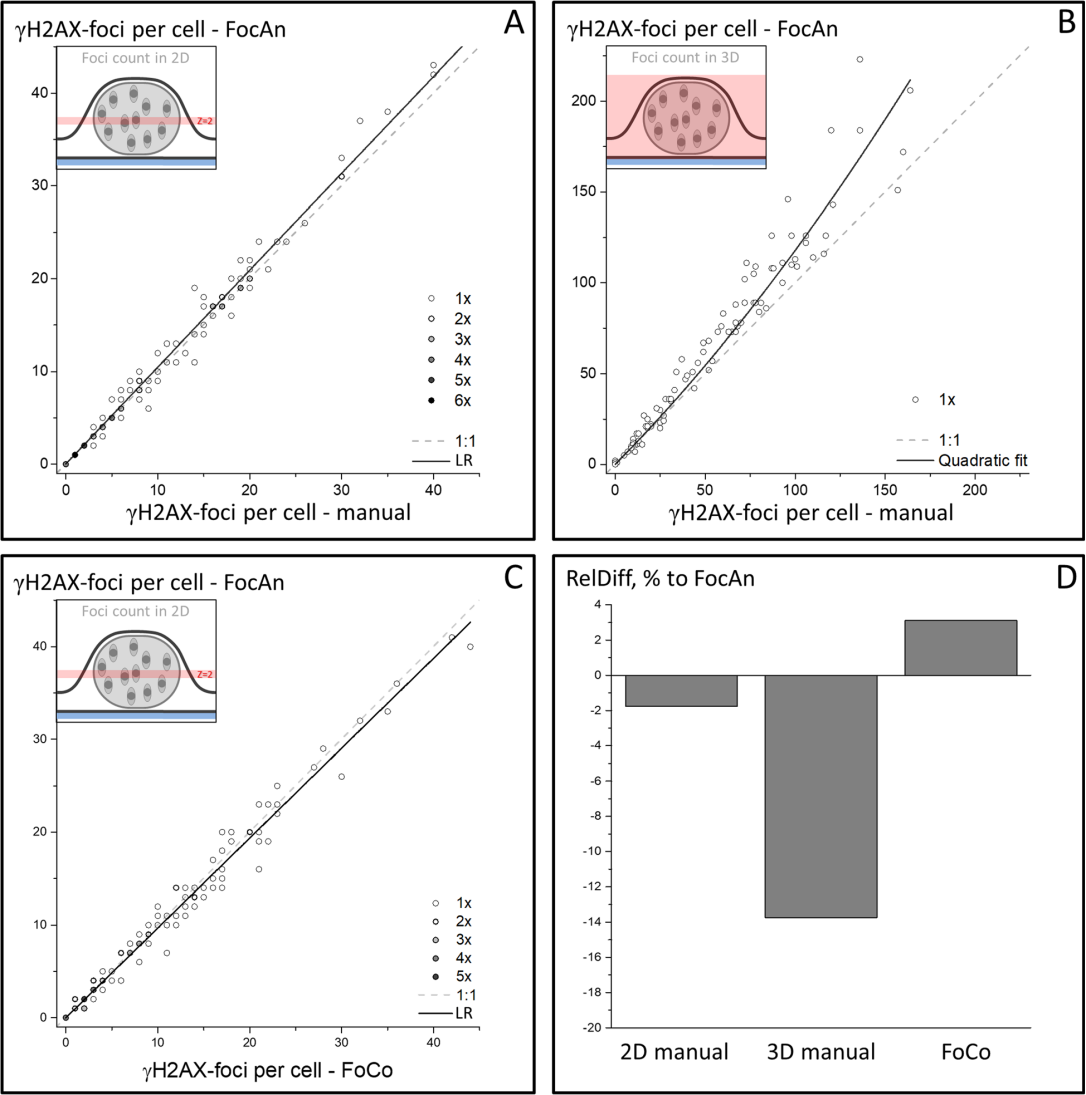
Comparison of FocAn-, FoCo- and manual foci counting in the same image data set, consisting of a random mixture of non-irradiated and irradiated (2 Gy) DK-MG and SNB19 cells (*N* = 100 cells). The insets in **a-c** depict the regions of interest (either midsection **a** and **c**, or whole nucleus **b**) in which foci were counted. The data acquired by FocAn was plotted against data of either a manual point-and-click approach (**a** and **b**) or FoCo-based data (**c**). The dashed lines in **a-c** illustrate ideal 1:1 relationships between the compared counting methods. The linear regressions to the data (solid lines in **a** and **c**) deviate only slightly from the 1:1 relation (for detail, *see* text). Comparison of the total 2D foci numbers (FN_2D_, **a** and **c**) also reveals little difference (~ 2–3%) between the applied methods (**d**). The 3D foci number per nucleus (FN_3D_) determined with FocAn exceeds the number of manually detected foci by ~ 14% (**b** and **d**). Moreover, with increasing foci number (i.e. FN_3D_ > ~ 50), FocAn yielded increasingly higher FN_3D_ values as compared to manual counting (**b**), as illustrated in (**b**) by the upwardly curved linear-quadratic fit (solid line) diverging from the 1:1 relationship (dashed line). The bars shown in (**d**) are relative differences in foci numbers with respect to those detected by FocAn, calculated as RelDiff = (FN-FN_FocAn_)/FN_FocAn_) × 100%

The second and third steps (Fig. 1b, c) serve to detect and separate the dim fluorescent nuclear area from even less fluorescent cytosol. This gradual signal separation is achieved by applying the ImageJ plugin “mean auto local threshold” (mean ALT, Fig. 2b) and the “Gaussian blur” ImageJ filter (Fig. 1c). The mean ALT transforms the weakly fluorescent cell areas to their binary estimates, in which the cell nuclei are already recognizable but not yet clearly discerned (Fig. 1b). Only after smoothing the binary image by Gaussian blurring with a sigma of ~ 10% of the nucleus diameter, the nuclear, cytosolic and extracellular areas became distinguishable from each other (Fig. 1c).

Next (Fig. 1, step D^1^), the mid-gray ALT (Eq. 2) is applied to create a binary mask with the locations and shapes of nuclei (Fig. 1d), discarding the signals form the cytoplasm and exterior. Thereafter, the binary mask is optimized by eliminating the remaining artifacts and by identifying individual nuclei using a combination of the basal cutoff, the 2D watershed and particle analyzer plugins. The adjustable basal cutoff is used to remove artifacts caused by potential imperfections on the glass surface made visible by the gradual signal separation approach (Additional file 2: Figure S2A). Since the depth of the basal cutoff depends on the sample slide tilt, a well leveled sample holder is recommended. The acquired 3D position data of the nuclei are z-projected, thus reducing the mask to two dimensions (Fig. 1d). Because the projected area is mainly defined by the midsection of the nucleus and not by its basal slices, the z-projection procedure is not affected by the above mentioned basal cutoff. In addition, the 3D-to-2D projection vastly reduces the processing time of the subsequent steps for nucleus identification. For the separation of converging nuclei, a 2D watershed approach with adjustable tolerance is used (Fig. 1d, red lines). In the last step of nuclei identification, the individual nuclei are detected using the particle analyzer plugin. This plugin enables the use of size exclusion in combination with a roundness dependent filter in order to exclude the artifacts at the periphery of the image (Additional file 2: Figure S2B, red arrows). The nuclei in contact with the image edge are also automatically discarded in order to exclude partially imaged nuclei from the analysis.

The immunostained γH2AX foci are identified by applying, in the first step, the median ALT (Eq. 3) with a domain radius of twice the mean foci diameter (here: r~ 30 px), which creates a 3D binary mask corresponding to each focus (Fig. 1e). Additionally, if selected, an adjustable 3D watershed (3DWS) approach for the separation of overlapping foci is performed using the 3D Image suite plugin [30]. The initial parameters (i.e. seeds) needed for the 3DWS approach are generated using a 3D maxima finder in the normalized unprocessed images (Additional file 1: Figure S1B). The 3DWS approach is computationally intensive and nearly doubles the overall computing time. Therefore it should only be activated to separate overlapping foci at high foci numbers per nucleus (Fig. 2b). The 3D Objects Counter plugin [31] is then applied to the cropped nuclei in order to detect and to count the foci in each individual nucleus.

The results for each nucleus are saved in the working directory as tab-delimited text files containing, among others, information about the number of foci per cell, surface area of the foci in μm^2^, the spatial coordinates, intensities of the identified foci and foci volume in μm^3^. Images can be saved for verification of the global watershed as binary *.tif files (Fig. 1d). Verification of the foci parameters can be performed with composite images containing the original image (Fig. 1a) combined with either 3D objects (i.e. foci) or surface maps of the foci (Fig. 1f). Nuclear regions of interest (ROIs) are saved as zip files and a binary map per stack (Fig. 1d) as a *.tif file. In addition, a composite image stack of the foci ROI (red) and the signal of foci (gray) can be saved as a *.tif file (Additional file 1: Figure S1A).

#### Image quality requirements for FocAn

FocAn uses 3D grayscale single color or multicolor image stacks, although only one channel (selected by the user, Additional file 1: Figure S1C) can be processed per run. During image acquisition, pixel saturation and very bright background objects, such as impurities and/or bacteria, have to be avoided. Also the laser intensity and pixel dwell time should be optimized for the fluorophore used in order to reduce photobleaching during acquisition of large image stacks. The use of BioFormats importer allows the processing of various common life science and setup specific image formats, including, but not limited to, TIF, TIFF, PNG, CZI, NEF, etc. Image parameters, such as voxel size, threshold domain radius and foci size estimates, are prompted in the first dialog window of the GUI (Additional file 1: Figure S1A).

#### Hardware requirements

FocAn can be run on all modern desktop workstations with installed ImageJ. However, it is recommended to use a 64-bit system with a multicore CPU for computationally intensive multithreaded local thresholding operations. Since the optional 3DWS plugin used is incapable of multithreading, a fast CPU (MHz) is also recommended. For example, a Quadcore i7–4790 CPU@3.6GHz needs ~ 4 min without and ~ 8 min with 3DWS segmentation to process a 1024 × 1024 × 60 image stack.

#### Additional ImageJ plugins used

3D Objectscounter v.2.0.1 in FIJI.

BioFormatsimporter v5.5.2 (included in FIJI).

Adj Watershed.

3D Imaging suite v3.9.

AutoLocalThreshold 1.16.5.

#### Critical issues and troubleshooting

Extremely bright objects, such as very dense clusters of antibodies, or occasional bright impurities should be avoided during image acquisition. Otherwise, the picture normalization can fail and compromise the nuclei recognition. The bright signals outside of the nuclei should be blacked out manually before analysis. The stand-by mode of MS Windows may interfere with the analysis of large batches of image data. The automatic stand-by function of MS Windows should be deactivated. During processing, clicking on any open ImageJ window should be avoided, because this can interfere with the call-up processes of the plugin and produce a critical failure.

## Results

### Comparison of automated foci counting with FocAn to manual foci counting

We first validated the algorithm implemented in FocAn by comparing the numbers of γH2AX foci detected automatically by FocAn to those obtained by manual foci counting in 2D images and 3D image stacks. To this end, we conducted in parallel automated and manual analyses using the same sample of GBM cells (*N* = 100) consisting of a random mixture of non-irradiated and irradiated (2 Gy) DK-MG and SNB19 cells at various time intervals after irradiation. Foci were counted either within the midsection plane (2D) of the nucleus, corresponding to the slice located ~ 3 μm above the glass slide, or slice-by-slice within the whole nuclear volume (3D), as illustrated in the insets of Fig. 2a and b, respectively. Manual foci counting was performed using the on-screen point-and-click method. Manual counting was carried out by two experienced operators who independently generated results from the same images/image stacks. The inter-operator foci counting results were very similar (coefficient of variation < 10%) with Pearson correlation coefficients of 0.987 and 0.998 for 2D and 3D data, respectively. The corresponding concordance correlation coefficients, ρ_c_ [32], were 0.987 and 0.994.

In Fig. 2a, the foci numbers per nuclear midsection (FN_2D_) detected with FocAn are plotted against the corresponding manually acquired values along with the best linear fit to the data (solid line). For computation of FN_2D_ values the 3D FocAn algorithm was modified to accept the 2D nuclei maps and the 2D data sets for foci detection. The foci number per nuclear midsection varies over a wide range within the analyzed sample, i.e. 0 ≤ FN_2D_ ≤ 42 foci/nucleus, as detected with FocAn. Judging from the slope of the regression line (~ 1.04 ± 0.007), the Pearson correlation coefficient (*r* = 0.997) and the concordance correlation coefficient (ρ_c_ = 0.992), the results obtained by two scoring methods agree very well over the whole data range shown in Fig. 2a.

As expected, the 3D foci numbers detected with FocAn in the whole nuclei (0 ≤ FN_3D_ ≤ 250 foci per nucleus, Fig. 2b) exceeded by far the corresponding data for the nuclear midsections (Fig. 2a). As also seen in Fig. 2d, the total foci number per whole nucleus (FN_3D_) determined with FocAn exceeds the number of manually detected foci by ~ 14%. Moreover, with increasing foci number per nucleus (i.e. FN_3D_ > ~ 50), FocAn yielded increasingly higher FN_3D_ values as compared to manual counting. This point is illustrated in Fig. 2b by the upwardly curved fitted line lying above the ideal 1:1 relationship between the two counting methods (dashed line in Fig. 2b). As a result, both the Pearson correlation coefficient (*r* = 0.970) and the concordance correlation coefficient (ρ_c_ = 0.928) deviate significantly from unity with increasing foci number per nucleus.

### Comparison of FocAn to FoCo

For further validation of the FocAn algorithm, we compared the results of foci counting obtained with FocAn and the open-source software FoCo [13], recently developed for 2D analysis of γH2AX foci. Unlike FocAn, which recognizes nuclei due to their dim background fluorescence, FoCo requires specific DNA staining with DAPI for nuclei recognition. Therefore, in order to apply FoCo to our images with unstained nuclei, we first extracted the midsection slice of the nucleus from the 3D image stacks used by FocAn. Since our raw images do not include specific nuclei staining, we also provided FoCo with the corresponding 2D binary maps of the nuclei (Fig. 1d). The 2D nuclei maps were generated from the raw 3D stacks using the FocAn algorithm, which was necessary because the gradual signal separation approach (Figs. 1a-c) implemented in FocAn for nuclei detection operates most precisely with 3D nuclei information. To ascertain that the comparison between FocAn and FoCo was not biased by the FocAn-generated nuclei input, we additionally proved the nuclei separation by hand using the generated nuclear ROIs and the corresponding raw images. The resulting images, with 2D foci in the red channel and 2D binary nuclei maps in the green channel, meet the FoCo requirement for multichannel 2D images. Analysis by FoCo was performed as described in [13], yielding the FN_2D_ numbers.

The corresponding FocAn-based FN_2D_ values were computed with the FocAn algorithm, which had been modified to accept the 2D nuclei maps and the 2D data sets for foci detection. In Fig. 2c, the foci numbers per nucleus midsection (FN_2D_) detected with FocAn are plotted against the corresponding FoCo-based data along with the best linear fit to the data (solid line). The calculated regression slope (~ 0.97 ± 0.01), the Pearson correlation coefficient (*r* = 0.995) and the concordance correlation coefficient (ρ_c_ = 0.988) indicate good agreement between the 2D foci counts obtained with FoCo and FocAn over the whole data range, with only an about 3% difference between the two methods (Fig. 2d).

### Automatic γH2AX foci counting in irradiated GBM cells

**Fig. 3:**
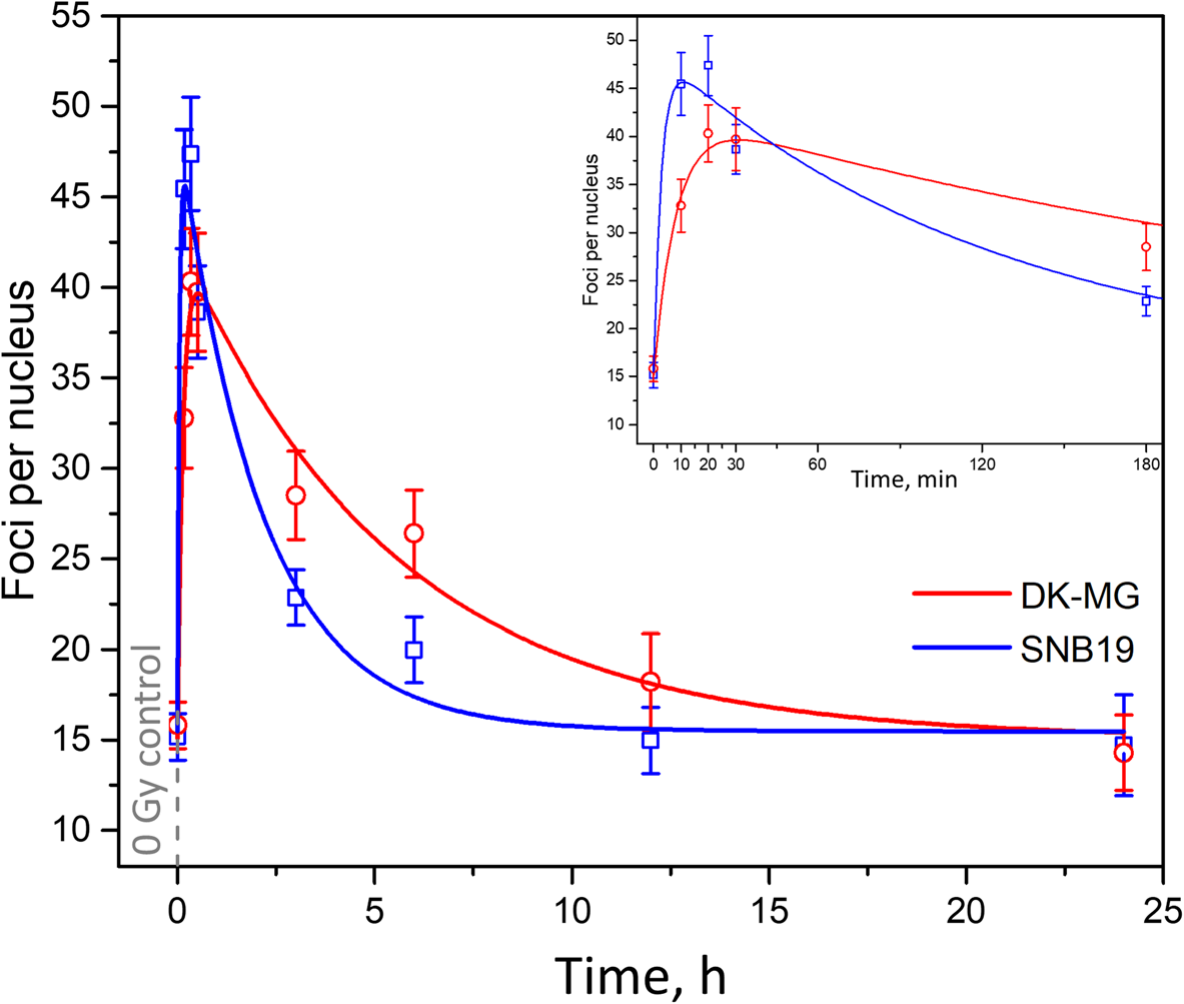
Time-courses of DNA DSB induction and repair in two glioblastoma cell lines, DK-MG and SNB19 (red and blue symbols, respectively). The cells were irradiated with 2 Gy, fixed at the indicated time intervals after irradiation, immunolabeled for γH2AX and examined by 3D confocal microscopy. Each data point represents the mean (±SE) foci number per nucleus of at least 80 cells. The 3D foci numbers were acquired automatically from the image stacks using FocAn. The total computation time for the depicted data was ~ 30 h. The inset shows γH2AX foci counts during the first 3 h after irradiation in detail. The lines are best fits of the modified Mariotti-model (Eq. 4; for detail see text and [33]) to the experimental data

The FocAn algorithm was applied to count foci in two different GBM cell lines (DK-MG and SNB19) irradiated with 2 Gy. The cells were fixed at various time intervals (up to 24 h) after irradiation, stained for γH2AX and examined by confocal microscopy. The images were then analyzed using FocAn and the foci counts were plotted against the repair time (Fig. 3). The mock irradiated controls (0 Gy) served as the initial points (*t* = 0). As seen in Fig. 3, the foci numbers in both cell lines exhibited two-phase kinetics (induction and decay) after irradiation. In SNB19 cells, the foci number grew rapidly from the value of ~ 15 foci per non-irradiated control nucleus to its maximum of ~ 47 foci/nucleus counted 10–20 min after irradiation (Fig. 3, blue symbols). After that, the amount of γH2AX foci decreased exponentially with time and reached the initial background value ~ 12 h after irradiation.

Compared to SNB19 cells, DK-MG cells displayed a slower foci induction kinetics with the peak value of ~ 40 foci/nucleus measured 20–30 min after irradiation. The foci decay in DK-MG cells occurred much slower than in SNB19 cells and required ~ 24 h to reach the background value of 16 foci/nucleus (red symbols in Fig. 3).

The mean foci counts obtained by FocAn were fitted to the modified model proposed by Mariotti et al. [33], which describes the dynamics of γH2AX foci numbers in irradiated cells (Eq. 4):

4 $$ N(t)={A}^2\left(1-{e}^{-t/{\tau}_1}\right)\left({e}^{-t/{\tau}_2}\right)+R $$

where the exponential terms $A\left(1-{e}^{-t/{\tau}_1}\right)$ and $A\left({e}^{-t/{\tau}_2}\right)$ describe the induction and decay processes of γH2AX foci, respectively. The unknown parameters, including the magnitude (*A*), the time constants of induction (τ_1_) and decay (τ_2_), as well as the background factor *R* were derived by fitting Eq. 4 to the data. Factor *R* was introduced to account for the preexisting γH2AX foci commonly observed in cancer cells [34].

As seen in Fig. 3, the Mariotti model (curves) fits very well our experimental data (symbols) for both tested cell lines. We found that in both cell lines foci induction occurred much faster than foci decay, i.e. τ_1_ < < τ_2_. Moreover, comparison of the fitted τ_1_ values shows that the foci induction rate in SNB19 cells (τ_1_ ≈ 2.8 min) was much higher than in DK-MG cells (τ_1_ ≈ 8.4 min). The difference in τ_1_ between the cell lines is particularly evident in the inset of Fig. 3. Despite similar initial DNA damage in both cell lines, SNB19 cells were able to repair DNA DSBs much faster (τ_2_ ≈ 125 min) than DK-MG cells (τ_2_ ≈ 326 min).

The total processing time to generate the data presented in Fig. 3 was about 18 h, using an ordinary computer, e.g. a Quadcore i7–4790 CPU@3.6GHz, which needs ~ 4 min without and ~ 8 min with 3DWS segmentation to process a 1024 × 1024 × 60 image stack. Each of the eight data points per cell line in Fig. 3 represents ~ 80 cell nuclei, which yields ~ 1280 nuclei evaluated in 3 dimensions. Each 3D image stack per nucleus consists of ~ 60 images. The total number of analyzed 2D images was therefore ~ 76,800. Manual evaluation of such a large number of images would obviously be unfeasible.

## Discussion

In this study, we developed a new algorithm (FocAn) for automatic counting of γH2AX foci in cell nuclei using confocal 3D image stacks. Unlike previous 2D approaches [11, 13] confined only to foci located within the midsection plane of the cell nucleus, FocAn enables the detection and quantification of the total number of foci distributed over the entire nuclear volume.

A further advantage of FocAn is that it does not require additional staining of cell nuclei with DNA staining dyes, commonly used for nuclei recognition [10–15]. Instead, FocAn uses the gradual signal separation approach (Figs. 1b-d) to detect and separate individual overlapping nuclei. Our approach relies on the faint unspecific signal from the nucleus. This feature not only simplifies staining protocol but also opens the opportunity to study an additional target using dyes in the otherwise occupied spectral bandwidth. For multicolor image hyperstacks (x,y,z,c), a specific color channel of interest can be selected for foci analysis (Additional file 1: Figure S1C). It is not necessary to further convert or split multicolor image stacks for analysis.

Obviously the amount of overlapping foci can be expected to increase at higher foci densities. This complicates the computer-based recognition of individual foci in conventional 2D images and necessitates 2D watershed image transformations [10–15]. Unlike earlier algorithms such as FoCo, Focinator, FociCounter, etc. [12–15, 20], FocAn executes a 3D watershed approach, which utilizes local maxima in a 3D environment to create initial coordinates for computing the separation boundaries between individual foci. Based on the information from 3D image stacks, the 3D watershed might be more sensitive for foci separation than 2D watershed approaches [35]. In fact, FocAn is able to recognize up to about 250 γH2AX foci per nucleus (Fig. 2b).

Another important feature of FocAn is the normalization procedure. Fluorescence imaging techniques typically suffer from a marked signal drift due to photobleaching of fluorophores within the imaged volume, especially for large z-stacks with long exposure times. Photobleaching inevitably shifts the saturation threshold in subsequent images to lower intensities [36]. To minimize the negative impact of photobleaching on foci recognition, FocAn uses slice-by-slice image normalization in combination with ALT.

To verify the reliability of our FocAn algorithm, we compared the foci counts obtained with FocAn to the previously established software FoCo [13] and also to manual counting, for both 2D and 3D data (*see* Fig. 2). We found that the number of foci in the nuclear midsections (2D) detected with FocAn show very high statistical correlations with manual counting (Fig. 2a) and also with the results of automatic analysis performed with FoCo (Fig. 2c). Particularly, the 2D foci counts obtained with FocAn deviate from the manual (Fig. 2a) and FoCo-based data (Fig. 2c) only by 2–3% (Fig. 2d). For comparison, FoCo-based counting exceeded manually scored foci numbers by ~ 3.5%, with a Pearson correlation coefficient r = 0.994.

However, if we compare our FocAn counts in 3D with the manual counts, a much higher discrepancy of ~ 14% was observed (Fig. 2b). Moreover, the difference between the automated and manual data increases with increasing foci density (solid curve in Fig. 2b). The observed discrepancy between two counting methods may be due to two independent factors. These are, first, an underestimation of the actual foci number by manual counting in case of high foci density (i.e. high foci numbers per nucleus), and, second, excessive foci fragmentation by the 3D watershed plugin implemented in FocAn. Therefore, in order to prove the reliability of the 3D watershed transformation for foci recognition, we examined manually the 3D foci detected with FocAn (Additional file 3: Video S1). The video, in which the 3D raw image stack (Fig. 1a) is overlaid with the final 3D foci map generated with FocAn (Fig. 1f), demonstrates the robustness of the 3D watershed approach and the lack of excessive foci fragmentation, i.e. oversegmentation, which is a general problem of watershed transformation [37]. We can therefore conclude that the main reason for the observed discrepancy is an underestimation of the actual foci number by manual counting in case of high foci numbers per nucleus. This underestimation can result from closely spaced and overlapping γH2AX foci, which are difficult to evaluate visually.

To provide user-friendliness and to reduce operator bias, we tried to keep the FocAn user interface as simple as possible. The first window prompt of the GUI only asks for the image pixel sizes and the typical dimensions of foci and nuclei (Additional file 1: Figure S1A). These parameters are defined by the microscope setup and the imaged object. They are therefore known or easily determined by the operator. In addition, FocAn features can be further customized by using the advanced setup prompt window of the GUI (Additional file 1: Figure S1B). Here, in-depth parameters can be changed, such as noise reduction options including the additional Gaussian blurring and a despeckle filter, which are recommended for images of poor-quality samples with weak fluorescence signals. However, additional blurring of the original images is usually not required as it yielded no noticeable difference for the detection of nuclei and foci. Direct modification of threshold levels and 3D watershed parameters, including initial parameters and size exclusion for nuclei and foci, are also included. The threshold domain radius for nucleus detection (given by the Adj Watershed ImageJ Plugin) can be set to a value as low as ~ 0.5 but it should not be lowered further in order to avoid false nucleus segmentation. In combination, these advanced options enable experienced users to analyze additional proteins forming foci or clustered structures in the nucleus. Also in-depth parameters can be modified to adapt the FocAn for the images obtained with other microscopy techniques.

## Conclusion

The here introduced FocAn algorithm represents a fast and efficient tool for the high-throughput-quantification of DNA DSB foci. It enables a user-independent 3D image analysis, capable of separating overlapped foci and detecting cell nuclei without additional nuclei staining. The application of image normalization in combination with different local thresholding algorithms compensates variations in signal and background intensity as well as in sample quality. The algorithm is implemented with the public domain ImageJ software and is freely available at https://sourceforge.net/projects/focan-3d/files/.

## Availability and requirements

**Project Name:** 3D Foci Analyzer.

**Project homepage:** https://sourceforge.net/projects/focan-3d/files/

**Operating system(s):** tested under MS Windows.

**Programming language:** Java; Ij1 Macro.

**Other requirements:** ImageJ v1.51 or above.

**License:** GNU General Public License version 3.0.

**Any restrictions to use by non-academics:** see license.

## Supplementary information

**Additional file 1: Figure S1.** Graphical user interface (GUI) of FocAn. The first prompt (Window **A**) inquires parameters for pixel-size calibration and crude foci specifications necessary for the auto local thresholding and segmentation parameters. The optional advanced setup options prompt (Window **B**) is for experienced users to activate or modify in-depth variables of noise suppression, 3D watershed and segmentation processes. The multicolor prompt (Window **C**) inquires the image channel containing the γH2AX foci information.

**Additional file 2: Figure S2.** Artefacts caused by the gradual signal separation approach. Image **A** shows possible artefacts due to the glass surface in the basal portion of the image stacks, roughly slices 1–10. Image **B** displays artefacts (indicated by red arrows) in the image edges, which are neglected by the algorithm.

**Additional file 3: Legend to Movie S1**. Slice-by-slice representation of the 3D image stack of γH2AX foci in four partially overlapping nuclei presented in Fig. 1. The main portion of the video displays the normalized γH2AX signals (gray, Fig. 1a) merged with the detected foci areas (red, Fig. 1e). The four insets on the right side of the video illustrate the γH2AX foci detected in the nuclei 1, 2, 3 and 4 from Fig. 1d

## Data Availability

All data generated and/or analyzed during this study are included in this published article and its Additional files and are available from the corresponding author on reasonable request.

## Abbreviations

3DWS: Three dimensional watershed
DSB: Double-strand break
ROI: Region of interest
