# Supplementary figures and images for: FocAn: automated 3D analysis of DNA repair foci in image stacks acquired by confocal fluorescence microscopy
Source: BMC Bioinformatics. 2020 Jan 28;21:27. doi: 10.1186/s12859-020-3370-8 (PMC6986076; doi:10.1186/s12859-020-3370-8)

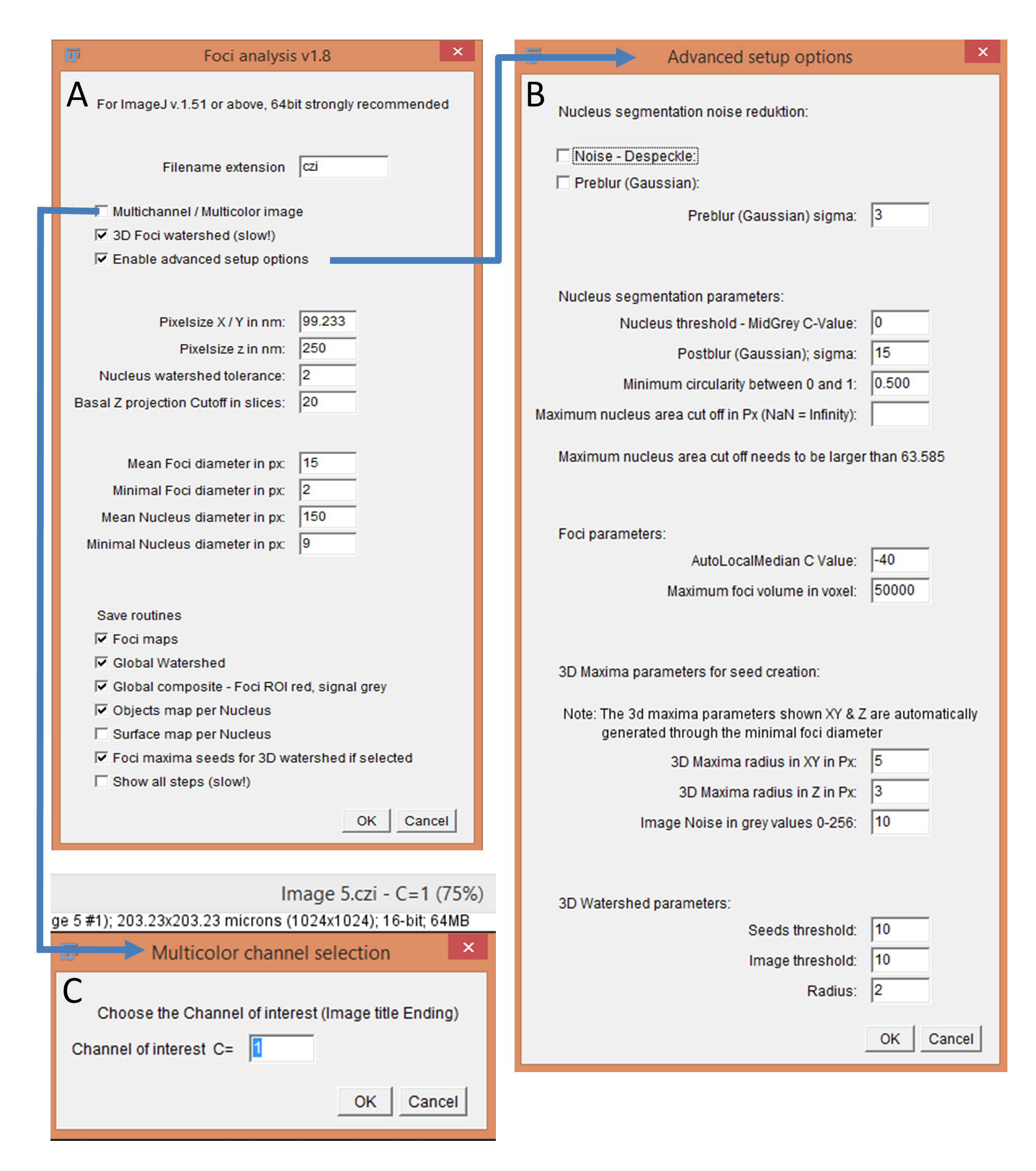

Supplement: Supplementary file 1 — Additional file 1: Figure S1. Graphical user interface (GUI) of FocAn. The first prompt (Window A) inquires parameters for pixel-size calibration and crude foci specifications necessary for the auto local thresholding and segmentation parameters. The optional advanced setup options prompt (Window B) is for experienced users to activate or modify in-depth variables of noise suppression, 3D watershed and segmentation processes. The multicolor prompt (Window C) inquires the image channel containing the γH2AX foci information. [file 12859_2020_3370_MOESM1_ESM.tif]

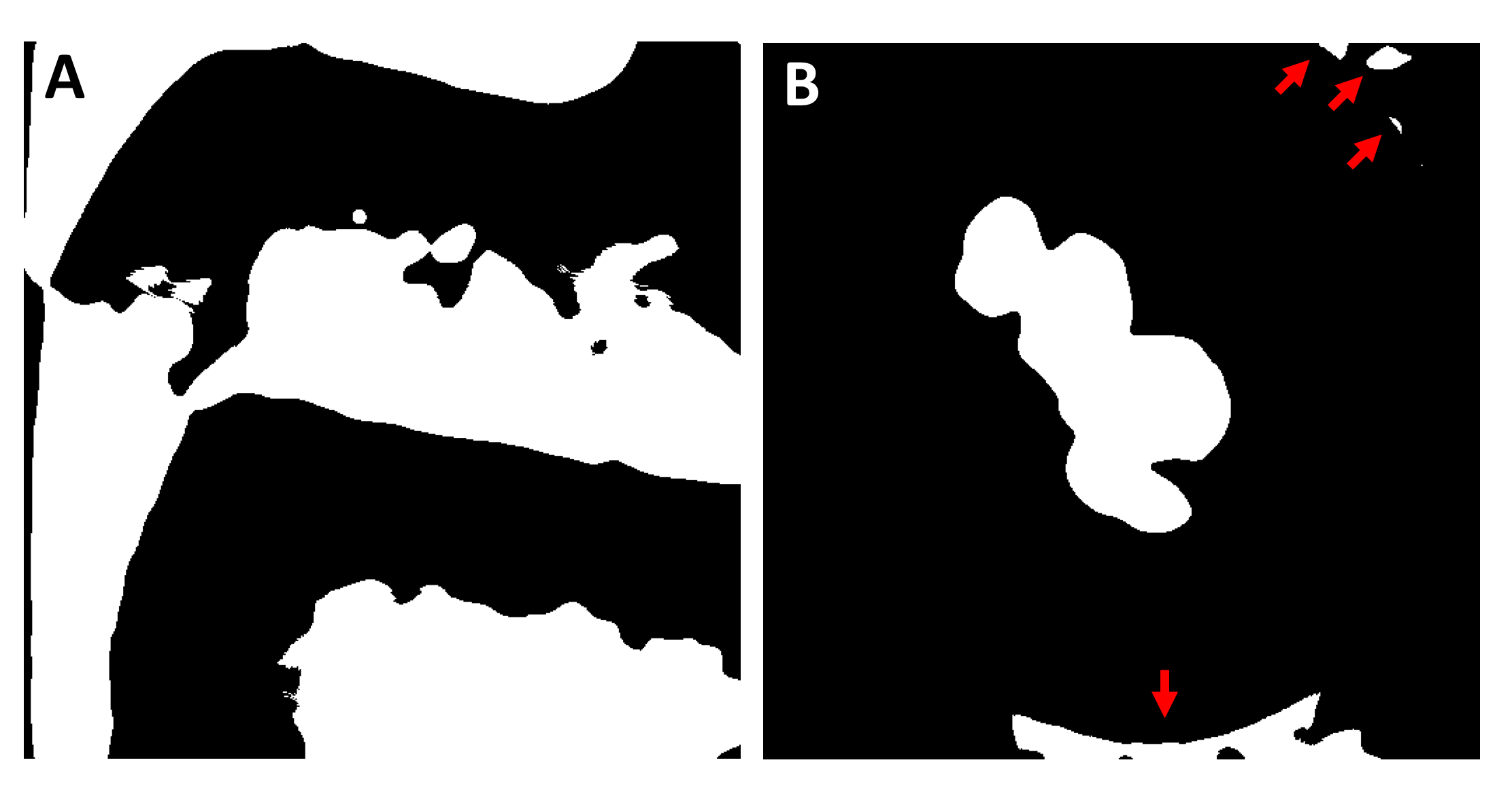

Supplement: Supplementary file 2 — Additional file 2: Figure S2. Artefacts caused by the gradual signal separation approach. Image A shows possible artefacts due to the glass surface in the basal portion of the image stacks, roughly slices 1–10. Image B displays artefacts (indicated by red arrows) in the image edges, which are neglected by the algorithm. [file 12859_2020_3370_MOESM2_ESM.tif]
